# A Bioinformatics Analysis of the Potential Roles of Aquaporin 4 in Human Brain Tumors: An Immune-Related Process

**DOI:** 10.3389/fphar.2021.692175

**Published:** 2021-05-25

**Authors:** Shuang Zou, Yu-Long Lan, Tong Ren, Xiangyu Li, Lijun Zhang, Hongjin Wang, Xun Wang

**Affiliations:** ^1^ Department of Neurosurgery, The Third People’s Hospital of Dalian, Dalian Medical University, Dalian, China; ^2^ Department of Neurology, Second Affiliated Hospital, School of Medicine, Zhejiang University, Hangzhou, China; ^3^ Department of Physiology, Dalian Medical University, Dalian, China; ^4^ Department of Neurosurgery, Second Affiliated Hospital, School of Medicine, Zhejiang University, Hangzhou, China; ^5^ Department of Breast Cancer, Key Laboratory of Breast Cancer Prevention and Therapy, National Clinical Research Center for Cancer, Tianjin’s Clinical Research Center for Cancer, Tianjin Medical University Cancer Institute and Hospital, Tianjin, China; ^6^ Department of Ophthalmology, The Third People’s Hospital of Dalian, Non-Directly Affiliated Hospital of Dalian Medical University, Dalian, China; ^7^ Department of Neurology, The Second Affiliated Hospital of Dalian Medical University, Dalian, China

**Keywords:** aquaporin 4, human brain tumors, tumorigenesis, immunoinformatics, CD8 + T-cell

## Abstract

Aquaporin 4 (AQP4) is an ubiquitously expressed membrane protein channel found in the central nervous system and mainly on astrocytes. Recent studies on AQP4 has implicated it in tumorigenesis. It is of interest to determine the potential value of AQP4 in identifying, guiding treatment and prognosticating various types of CNS cancers. This investigation systematically investigated the oncogenic role of AQP4 across 33 CNS tumors found in GEO and TCGA datasets. We found that CNS tumors strongly expressed AQP4. There appeared to be a strong link between the prognosis of patients with a CNS malignancy and degree of AQP4 expression. AQP4 expression influences the degree of CD8^+^ T-cell infiltration level as well as cancer-associated fibroblast infiltration in CNS tumors. Moreover, synaptic vesicle cycle and phosphatidylinositol signaling system-associated functions were also found to be related to AQP4 functional mechanisms. Furthermore, potential AQP4 inhibitors have also been explored by using Specs data base and virtual screening technique. This study contributes toward current knowledge regarding the role of AQP4 in CNS tumors.

## Introduction

Recent literature highlights the central role of AQP4 in cancer initiation and progression (Papadopoulos and Saadoun, 2015; Lan et al., 2020; Zou et al., 2020). Understanding its roles in tumorigenesis and tumor development is indispensable knowledge that contributes toward the development of molecular-targeted therapies. The incorporation of AQP4 targeting is an overlooked and underdeveloped strategy in cancer therapy.

Our most recent review has confirmed that AQP4 is highly expressed in glioma tissues, and AQP4 exerted carcinogenic effects via various pathways in glioma (Lan et al., 2017). Furthermore, regulatory T-cell development has been found to be dependent on AQP4 expression (Chi et al., 2011). Mice lacking AQP4 receptors had suppressed levels of CD4^+^/CD25^+^ regulatory T-cells. This leads to an abnormally overactive microglial inflammatory response (Chi et al., 2011). A pan-cancer analysis is necessary for further exploring how AQP4 contributes toward maintaining immune homeostasis, in addition to its key function in tumorigenesis across human brain cancers.

Cancer is a multifactorial and complex disease. In order to better understand the disease progress, further analysis regarding certain genes of interest and their association to clinical progress is necessary. The publicly available TCGA and GEO database is a compilation of functional genomic datasets of various types of human cancers (Tomczak et al., 2015; Clough and Barrett, 2016; Blum et al., 2018), enabling several different and more thorough analyses to be made. In the current study, data extracted from both these databases allowed for a detailed scrutiny of the relationship between AQP4 and several types of CNS malignancies. We also explored gene expression and alteration, signaling pathways, immune infiltration, protein phosphorylation and overall patient survival to characterize the nature of AQP4 in the biology and clinical prognosis in patients with CNS cancers.

## Materials and Methods

### Gene Expression Analysis

We sought to compare the expression levels of AQP4 protein between tumor and non-tumor tissues. The web-based TIMER2 (tumor immune estimation resource, version 2) (http://timer.cistrome.org/) tool was used to demonstrate the expression profiles of AQP4 across tumors and normal healthy tissues as well as in tumor data available on the TCGA database (Yang et al., 2007). The HEPIA2 “Pathological Stage Plot” module was used to generate violin plots of AQP4 expression across various pathological stages of all tumors available on the TCGA database. The UALCAN portal (http://ualcan.path.uab.edu/analysis-prot.html) allowed for an analysis of protein expression of the CPTAC (Clinical proteomic tumor analysis consortium) dataset (Chen et al., 2019).

### Survival Prognosis Analysis

GEPIA2 were used to determine the DFS (Disease-free survival) and OS (Overall survival) information of all TCGA tumor data in relation to AQP4 expression (Tang et al., 2019). Cutoff-high (50%) and cutoff-low (50%) values were defined as thresholds in determining if the tumor was of the high-or low-expression group.

### Genetic Alteration Analysis

Using the tool on cBioPortal web (https://www.cbioportal.org/) [(Gao et al., 2013), (Cerami et al., 2012)], “TCGA Pan Cancer Atlas Studies” in the “Quick select” section was chosen and “AQP4” was set as the query of genetic alteration characteristics of AQP4. The “Cancer Types Summary” module depicted the CNA (Copy number alteration), mutation type and alteration frequency across all TCGA tumors. The “Mutations” module allowed us to gain information regarding mutated sites in the form of schematic or 3D protein structure diagrams. Data regarding the disease-free, progression-free and overall patient survival differences between those with or without an AQP4 genetic alteration across tumors in the TCGA database was derived from the “Comparison” module.

### Immune Infiltration Analysis

The TIMER2 web server “Immune-Gene” module was used to investigate the relationship between immune cell infiltration and AQP4 expression across tumors in the TCGA database. Cancer-associated fibroblasts and immune cells of CD8^+^ T-cells were selected. The EPIC, MCPCOUNTER, XCELL, QUANTISEQ, CIBERSORT-ABS, CIBERSORT and TIMER algorithms were used to estimate the degree of tumor immune cell infiltration.

After that, the CIBERSORT (https://cibersort.stanford.edu/index.php) was used to further explore the association between the expression of AQP4 and immune infiltrates across all TCGA tumors. Data of gene expression levels of GBM was downloaded from TCGA data base (https://gdc-portal.nci.nih.gov/), in which the Illumina HiSeq 2000 RNA Sequencing was used as the testing platform, and 153 GBM samples were finally included.

### AQP4-Related Gene Enrichment Analysis

The STRING website (https://string-db.org/) was explored utilizing the query of a single protein name (“AQP4”) and organism (“*Homo sapiens*”). Restrictions including the active interaction sources (“experiments”) and maximum number of interactors to show (“no more than 50 interactors” in first shell) were set. Using these methods, we were able to determine proteins that bind to AQP4.

The top 100 AQP4-correlated targeting genes was determined using the “Similar Gene Detection” module of GEPIA2. We also applied the “correlation analysis” module of GEPIA2 to carry out a pairwise gene Pearson correlation analysis of AQP4 and preselected genes. Heatmap data of selected genes was derived from the “Gene_Corr” module of TIMER2.

GO (Gene ontology) and KEGG (Kyoto encyclopedia of genes and genomes) pathway analyses were carried out on a composite of two datasets. Gene lists were uploaded to DAVID (https://david.ncifcrf.gov/) with the settings of species (“*Homo sapiens*”) and selected identifier (“OFFICIAL_GENE_SYMBOL”) in order to obtain a functional annotation chart. The “tidyr” and “ggplot2” packages of the R language software (R-3.6.3) (https://www.r-project.org/) were used to visualize the enriched pathways.

### Virtual Screening and Molecular Docking

The Spec database was used to predict potential AQP4 inhibitors. Molecular dockings of our own constructed 3D model of AQP4 (human) and potential inhibitors were performed. Homologous modeling of human AQP4 (PDBID: 3gd8) and potential inhibitors were carried out using the I-Tasser tool. The Schrodinger software (Glide module) was used to perform docking research. The pretreatment process of the Glide protein was as follows: Bhydrogenation > dehydration > protein structure optimization^. The Epik mode of Ligprep weas used to treat small molecules. The lattice file was then selected from the center of the ouabain, with the pocket representing the surrounding 15 Åresidue (the size was 15 Å × 15 Å × 15 Å). Glide was then used to obtain the docking conformation with the highest precision docking (XP) and the conformation with the highest score was selected for further analysis.

## Results

### Gene Expression Analysis Data

**FIGURE 1:**
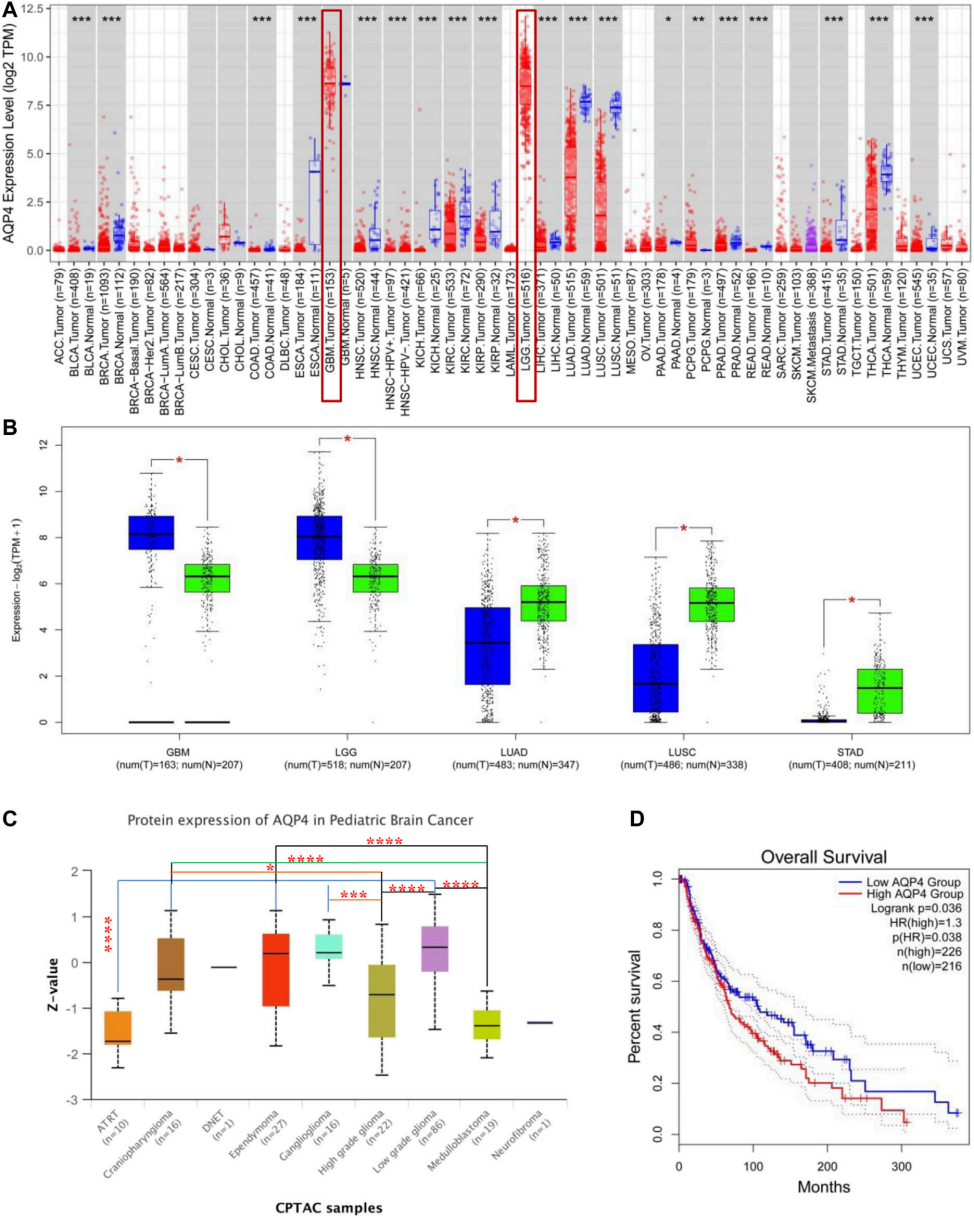
AQP4 gene expression across various tumors and pathological stages. **(A)** TIMER 2 was used to determine AQP4 expression across different cancers. *p < 0.05; **p < 0.01; ***p < 0.001. **(B)** TCGA database derived data on GBM, LGG, LUAD, LUSC and STAD cancers, with controls being corresponding normal tissues from the GTEx database. *p < 0.05 **(C)** AQP4 protein expression of normal tissue and pediatric brain cancer tissue from the CPTAC dataset was compared. **(D)** AQP4 gene expression and survival of cancer patients from the TCGA database was compared. The GEPIA2 tool was used to perform overall survival analyses of various cancers in TCGA based on expression of the SNRPG gene. The survival map and Kaplan-Meier curves are demonstrated. *p < 0.05; **p < 0.01; ***p < 0.001; ****p < 0.0001.

AQP4 expressions across different types of cancer data available on the TCGA was assessed using the TIMER2 approach. As depicted in Figure 1A, AQP4 expressions in PCPG (Pheochromocytoma and Paraganglioma) were significantly raised in contrast to the control tissues (*p* < 0.05); while the expression of AQP4 in UCEC (Uterine Corpus Endometrial Carcinoma), THCA (Thyroid carcinoma), STAD (Stomach adenocarcinoma), READ (Rectum adenocarcinoma), PRAD (Prostate adenocarcinoma), PAAD (Pancreatic adenocarcinoma), LUSC (Lung squamous cell carcinoma), LUAD (Lung squamous adenocarcinoma), LIHC (Liver hepatocellular carcinoma), KIRP (Kidney renal papillary cell carcinoma), KIRC (Kidney renal clear cell carcinoma), KICH (Kidney Chromophobe), HNSC (Head and Neck squamous cell carcinoma), ESCA (Esophageal carcinoma), COAD (Colon adenocarcinoma), BRCA (Breast invasive carcinoma) and BLCA (Bladder urothelial carcinoma) were notably lower in contrast to the corresponding control tissues (*p* < 0.05). AQP4 expressions were also assessed in tumor and normal tissue data available on the GTEx dataset, which included STAD, LUSC, LUAD, LGG (Brain Lower Grade Glioma) and GBM (Glioblastoma multiforme) (Figure 1B, *p* < 0.05). However, there were no significant differences for the other tumor data available on GTEx. Figure 1C demonstrates the AQP4 expression patterns in various brain cancer data available on the CPTAC dataset. Using the TCGA data, we also cohorted patients into having either low- or high-expression groups and investigated the association of AQP4 with patient prognosis. Higher AQP4 expressions were related to poorer patient prognosis as well as shorter OS (Survival) for LGG cancers within the TCGA project (Figure 1D).

### Genetic Alteration Analysis Data

**FIGURE 2:**
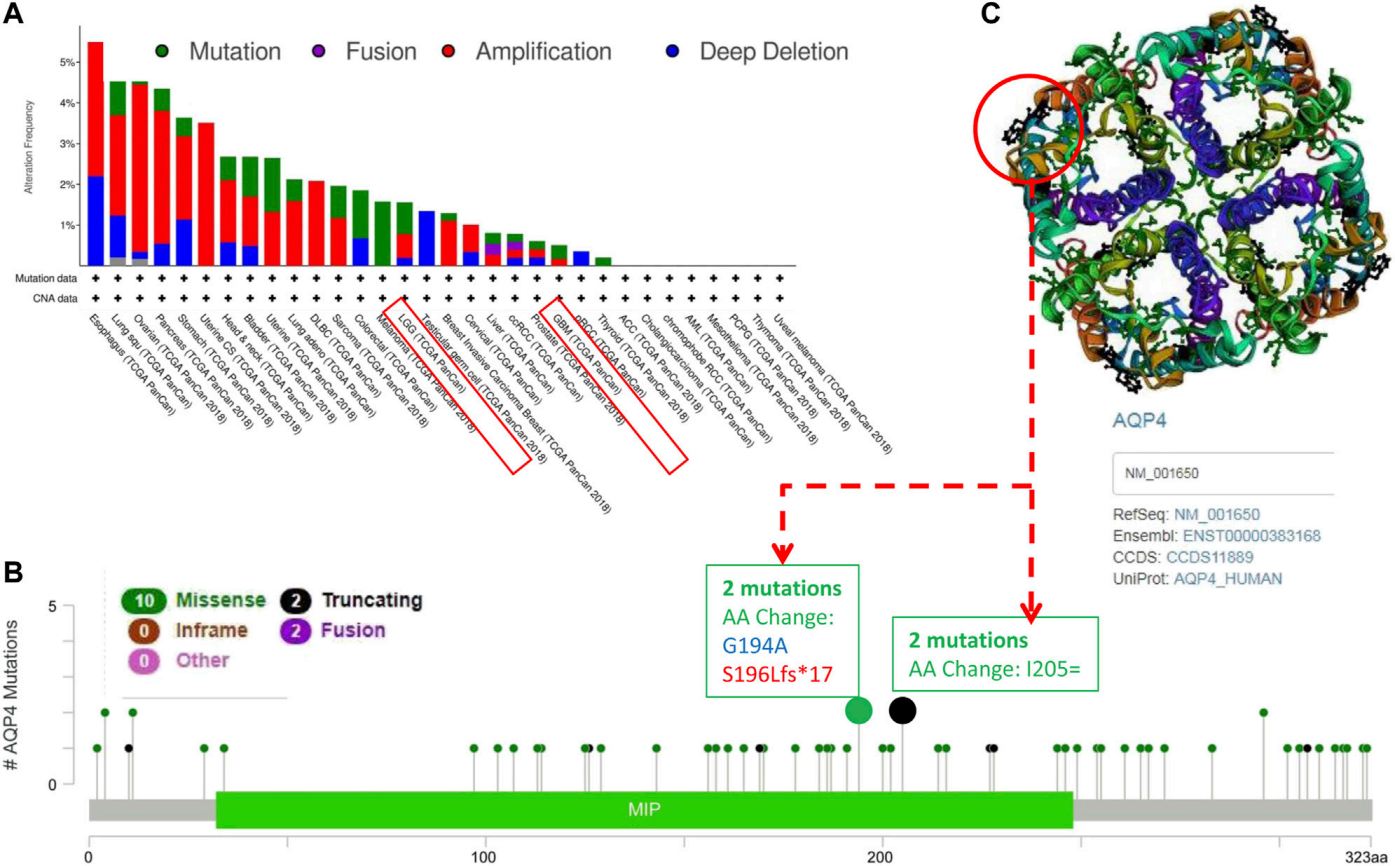
Mutation patterns of AQP4 across different cancers in the TCGA database. Using the cBioPortal tool, SNRPG mutations present in tumors in the TCGA database were analyzed. The alteration frequency with mutation type **(A)** and mutation site **(B)** are demonstrated. We display the mutation sites with the highest alteration frequency in the 3D structure of SNRPG **(C)**.

**FIGURE 3:**
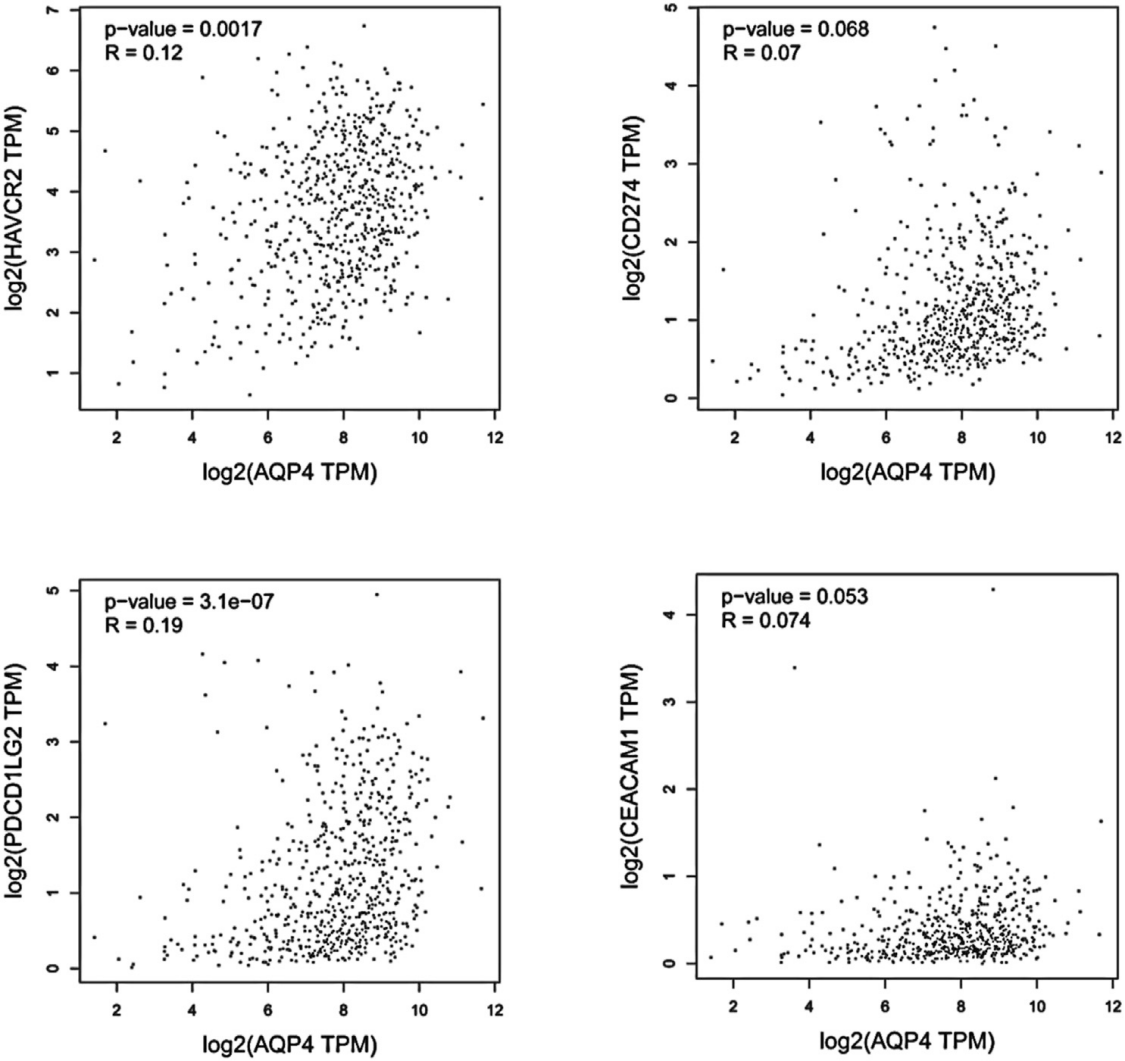
Using GEPIA, the expression correlation between AQP4 and AQP4-correlated immune checkpoints were analyzed, including TIM-3 (HAVCR2), PD-L1 (CD274), PD-L2 (PDCD1LG2), and CEACAM1, using GBM and LGG samples.

We further assessed the genetic alteration status of AQP4 in several tumor samples of the TCGA cohorts. Results indicated that patients with highest alteration frequency of AQP4 were those with esophagus tumors of the “amplification” and “deep deletion” subtypes (∼3% frequency) (Figure 2A). The “amplification” was the primary type for lung squ tumors, and similar results were also found for ovarian, pancreas, uterine CS, stomach, and DLBC tumors (Figure 2A). The “mutation” type of CNA was the primary type in the melanoma, which show an alteration frequency of ∼1.5%. The “deep deletion” CNA subtype was the primary type in the testicular germ cell cancer cases (Figure 3A). Interestingly, more than half of head and neck, bladder, uterine, lung adenocarcinoma and sarcoma cancer cases with genetic alteration (∼1% frequency) had copy “amplification” alteration (Figure 2A).

Figure 3B depicts the types, sites and case number of AQP4 genetic alterations. The most frequently encountered genetic alterations in the AQP4 gene were missense and truncation mutations. Among missense mutations, one AA change (G194A) alteration in the MIP domain was detected in 1 case of cancer, and one S196Lfs*17 alteration in the MIP domain was detected in 1 case of cancer (Figure 2B), which was able to induce amino mutations of the AQP4 gene at the 194 site of AQP4 protein, resulting in subsequent AQP4 protein truncation. Among truncation mutations, AA change (G194A) alteration in the MIP domain was detected in 2 cases of cancer. We further observed the 194 site in the 3D structure of AQP4 protein (Figure 2C). This result would benefit from further research.

### AQP4-Related Immune Checkpoints Analysis

We hypothesized that AQP4 could also be the regulatory factors of various immune checkpoints in brain tumors, such as TIM-3 (HAVCR2), PD-L1 (CD274), PD-L2 (PDCD1LG2), and CEACAM1. Thus, correlation analysis of AQP4 and various checkpoints expressions was then performed with given sets of TCGA expression data by using the Gene Expression Profiling Interactive Analysis (GEPIA) online tool (http://gepia.cancer-pku.cn/). These results indicated that AQP4 could be greatly related to various immune checkpoints in human GBM (*p* < 0.05) (Figure 3). Thus, we hypothesized that AQP4 may also be the key factor suppressing GBM malignancy via regulating immune checkpoints.

### Immune Infiltration Analysis Data

**FIGURE 4:**
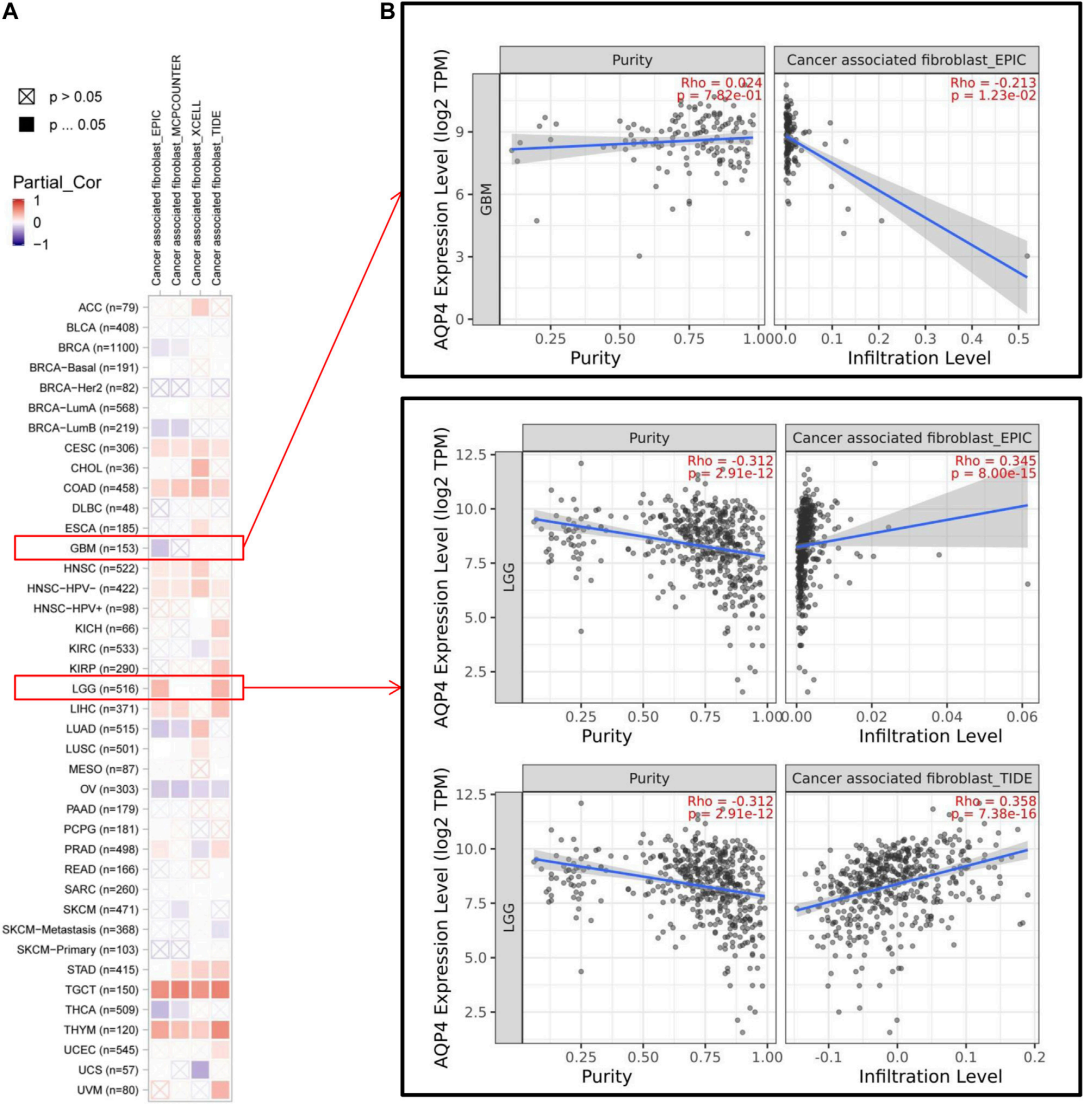
The correlation analysis between AQP4 expression and tumor cell infiltration of cancer-associated fibroblasts was performed using different algorithms across various types of cancer information from the TCGA database.

Cancer development, progression and metastasis have been found to be dependent on the degree of tumor immune cell infiltration (Saadoun et al., 2002). Stromal cancer-associated fibroblasts present in the tumor microenvironment have been found to exert a significant effect on various tumor functions (Warth et al., 2007; Ding et al., 2011). We then sought to determine the association between AQP4 gene expression and the degree of tumor immune cell infiltration across different cancer data available in TCGA using the TIMER, MCPCOUNTER, CIBERSORT-ABS, XCELL, CIBERSORT, QUANTISEQ and EPIC algorithms. AQP4 expression was noted to be significantly related to levels of cancer-associated fibroblasts of the CESC, CHOL, HNSC [HPV (Human papillomavirus) -], LIHC, STAD, TGCT and THYM cancers. However, OV cancers were found to be negatively associated to AQP4 expressions (Figure 4A). Figure 4B depicts scatterplot data of the above tumors produced using a single algorithm. AQP4 expression level in CESC correlates positive with the degree of cancer-associated fibroblast infiltration (Figure 4B, cor = 0.192, *p* = 1.33e-03) based on the XCELL algorithm.

**FIGURE 5:**
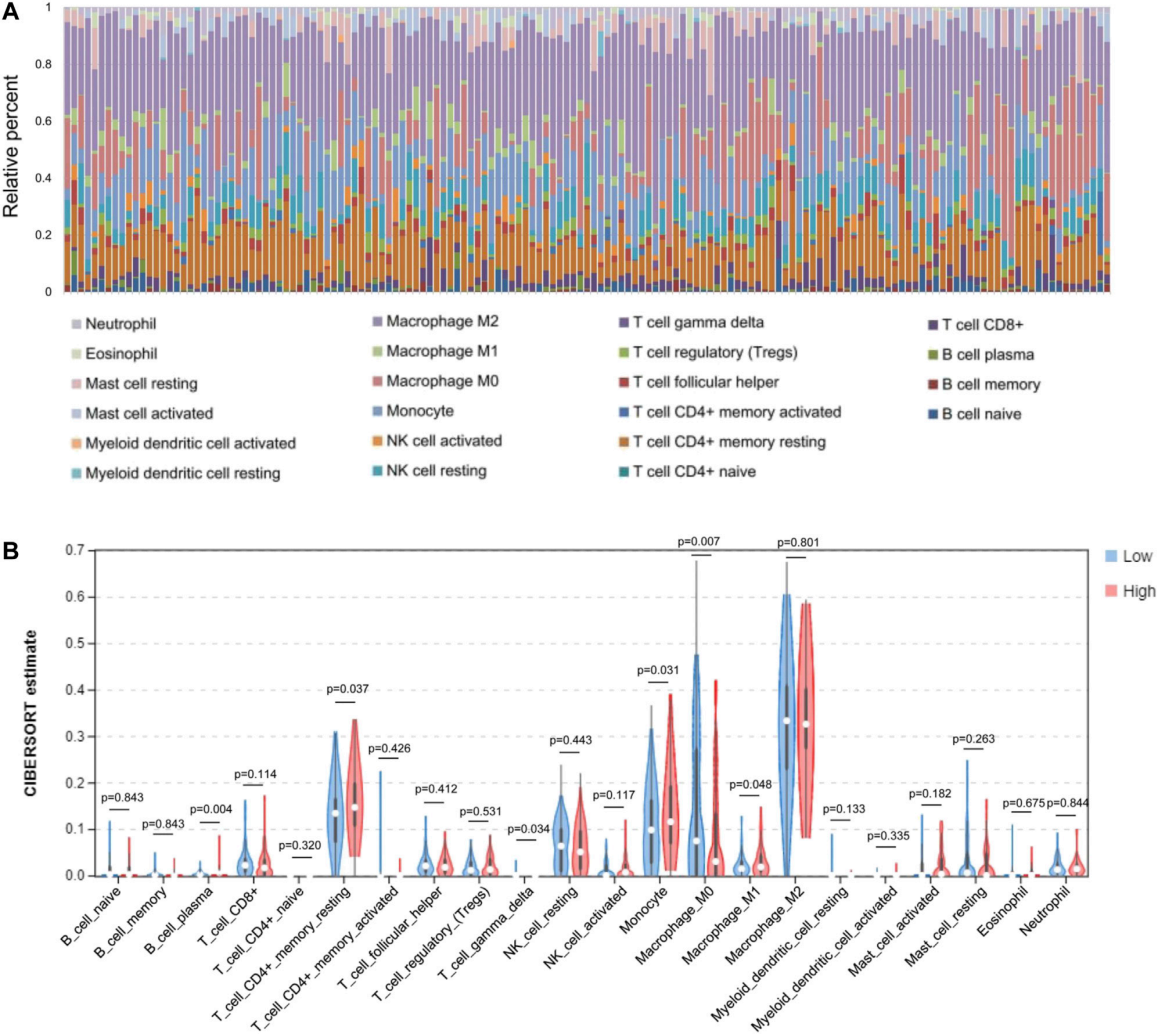
Comparisons of 22 important immune fractions between low- and high-AQL4 groups. **(A)** The specific 22 immune fractions represented by various colors in each sample were shown in barplot. **(B)** Wilcoxon rank-sum test revealed that the infiltration levels of CD8^+^ T cell, CD4^+^ memory resting T cell, M1 and M2 macrophages, as well as dendritic resting cells in high-AQP4 group were lower compared with that in low-AQP4 group.

Based on the expression levels of various GBM genes in the TCGA database, we used the CIBERSORT (https://cibersort.stanford.edu/index.php) tool to calculate the component ratio of 22 types of immune cells in each sample. And the composition of various immune cell types in each sample has been visualized (Figure 5A). After that, the samples were divided into low level (expression level below median) and high level (expression level above or equal to median) groups, according to the gene expression level of the AQP4. The proportional difference of various immune cells in the two different expression levels was analyzed between two groups (Figure 5B).

### Enrichment Analysis of AQP4-Related Partners

**FIGURE 6:**
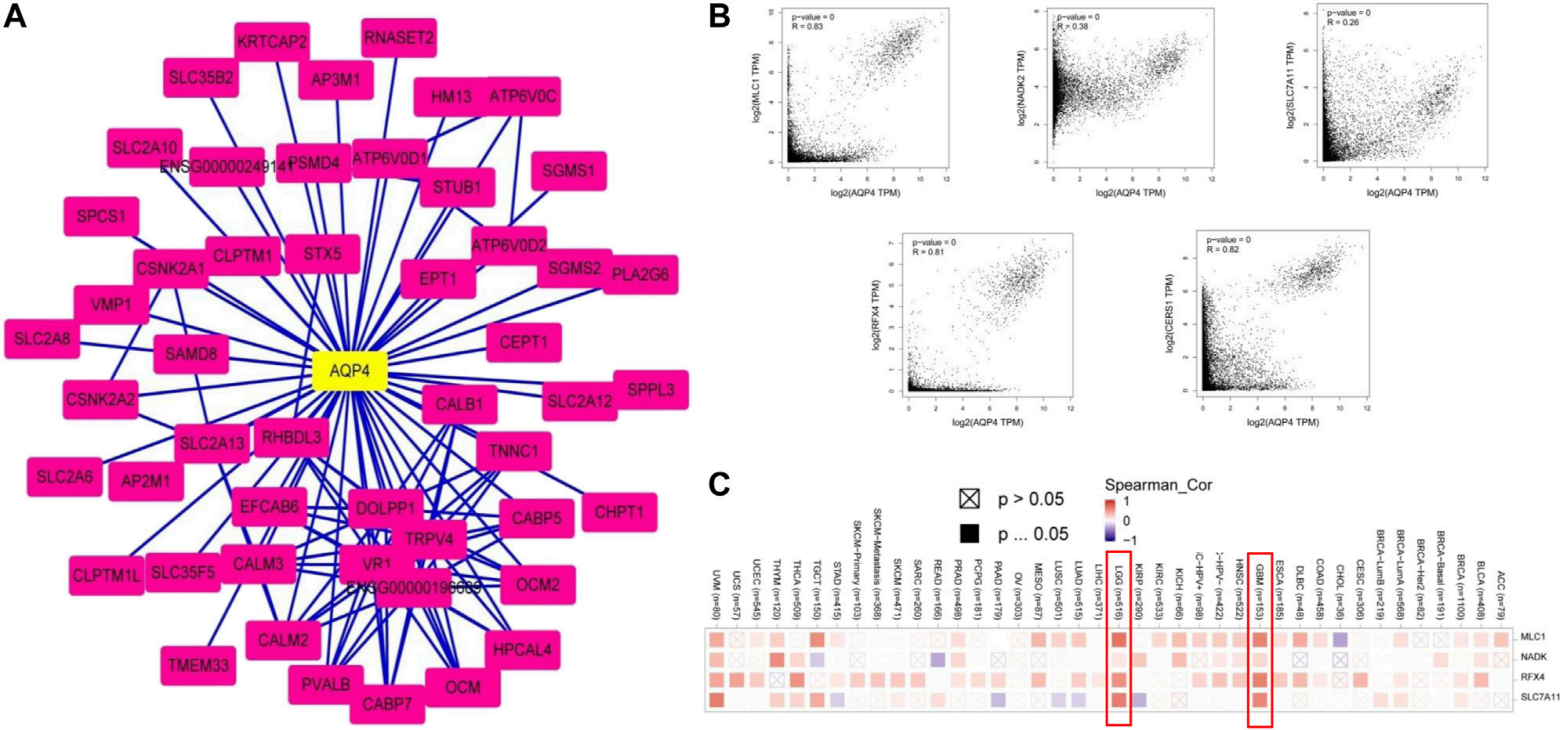
AQP4-related gene enrichment analysis. **(A)** Firstly, protein candidates which bound to AQP4 were predicted using the STRING tool. **(B)** The GEPIA2 allowed us to determine the top 100 AQP4-correlated genes from the TCGA database. The correlation between AQP4 expression and selected targeting genes were analyzed, including MLC1, NADK2, SLC7A11, RFX4, and CERS1. **(C)** The corresponding heatmap data based on detailed cancer types are also shown.

We then sought to uncover the role of the AQP4 gene in tumorigenesis. Potential AQP4 protein binding targets and genes associated to AQP4 expression were determined. The STRING tool yielded a total of 50 AQP4-binding proteins supported by experimental evidence. Figure 6A demonstrates the interaction network of these proteins. The GEPIA2 tool was used to combine all brain cancer tumor expression data in the TCGA databased and determined the top 100 genes that correlated with AQP4 expression. As shown in Figure 6B, the AQP4 expression level correlated positively to that of MLC1 (*R* = 0.83), NADK2 (*R* = 0.38), SLC7A11 (*R* = 0.26), RFX4 (*R* = 0.81), and CERS1 (*R* = 0.82) genes (all *p* < 0.001). There was also a positive association between AQP4 and the above five genes across various different cancers (Figure 6C).

**FIGURE 7:**
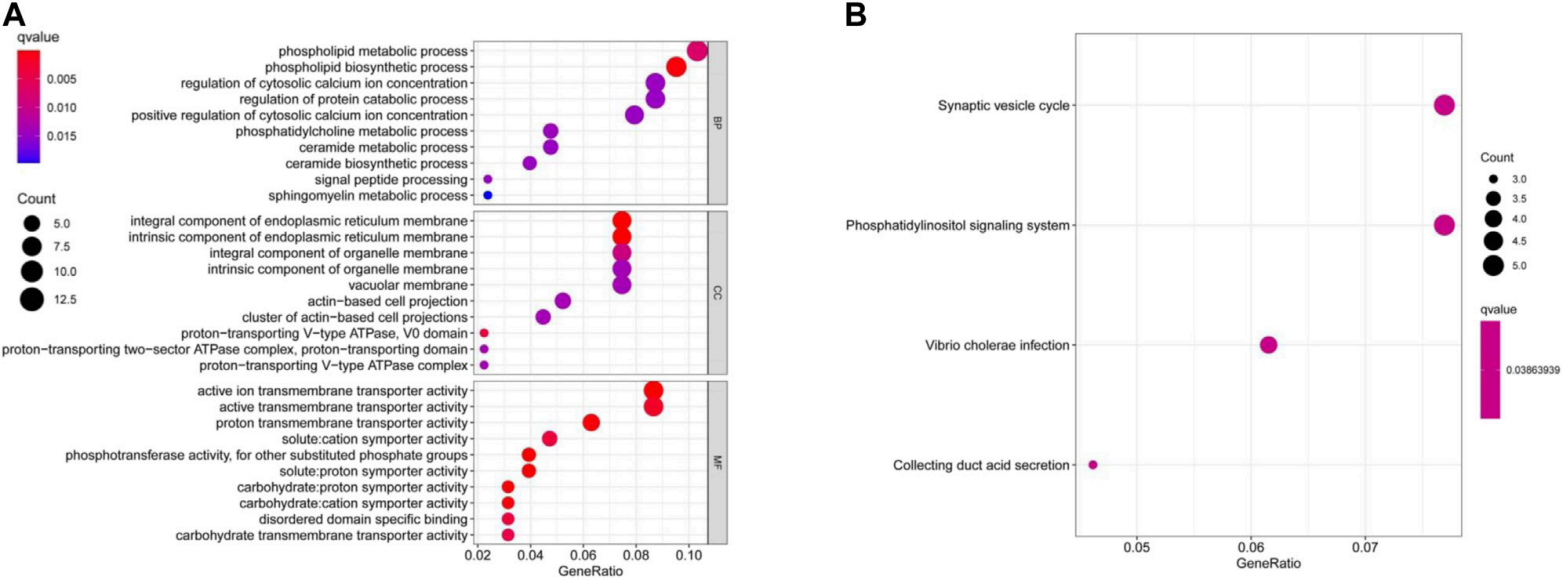
AQP4-related gene enrichment analysis. **(A)** GO analysis depicts molecular functional data of potential genes that may interact with AQP4 **(B)** KEGG analysis was also conducted.

GO and KEGG enrichment analyses were performed on two combined datasets. GO enrichment analysis data revealed that a majority of these genes were associated to pathways or cellular biology of active (ion) trans-membrane transporter activity and others (Figure 7A). The KEGG data suggested that “synaptic vesicle cycle” and “phosphatidylinositol signaling system” were mainly involved in AQP4 effects on tumor pathogenesis (Figure 7B).

### Virtual Screening of Potential AQP4 Inhibitors and Molecular Docking Analysis

**FIGURE 8:**
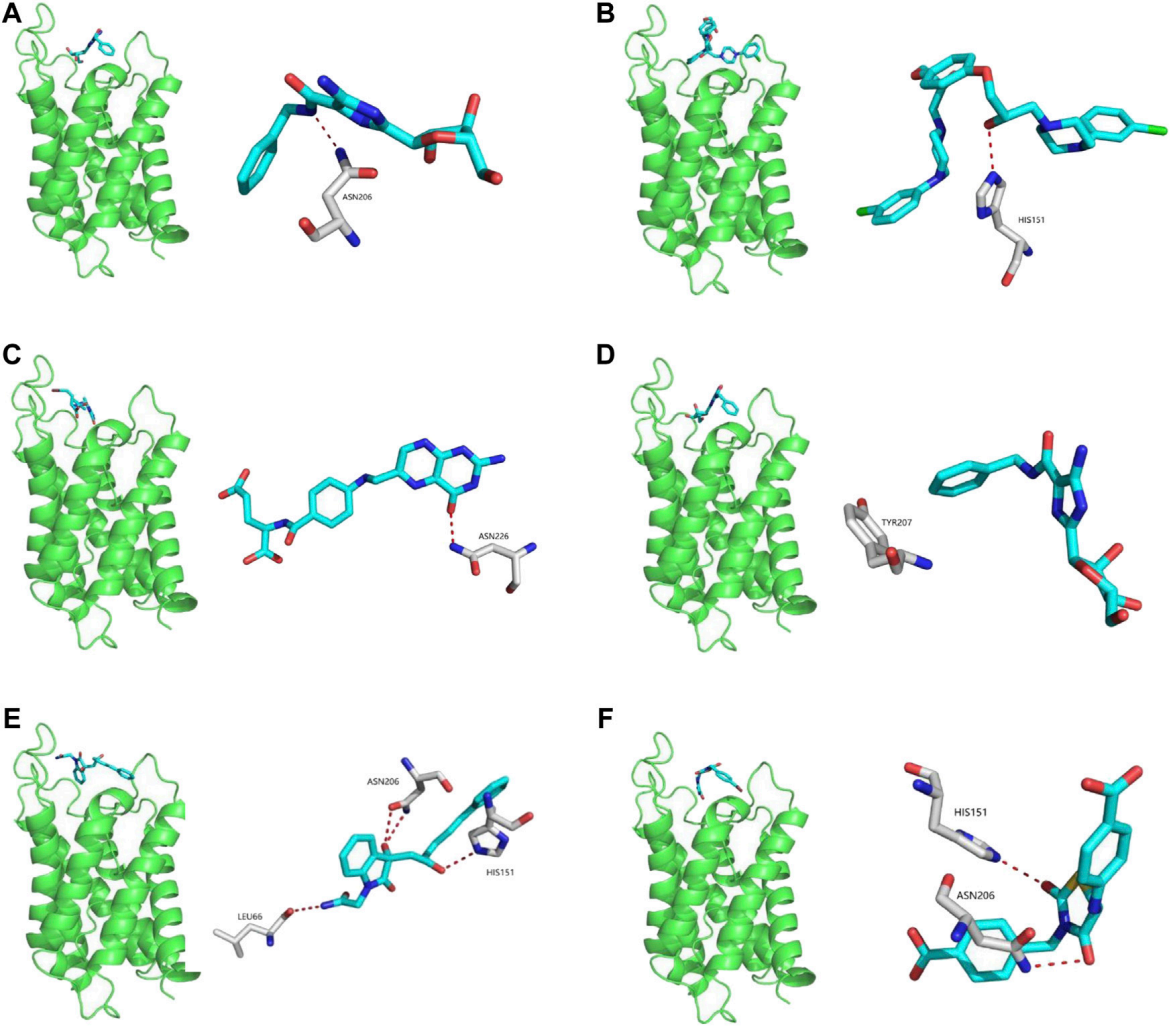
Virtual screening of potential AQP4 inhibitors and molecular docking analysis. The docking conformation of the top six small molecules with the modeling protein is provided and the docking mode and important residues are given.

Potential AQP4 inhibitors were explored using the Specs data base. We performed a virtual screening of the Specs data base using schrdinger software. The docking conformation of the top 20 small molecules with the modeling protein is demonstrated (Table 1), along with the docking mode and important residues (Figure 8). Recognition of AQP4-specific inhibitors may pave the way for the creation of novel molecular targeting agents useful in the treatment of CNS cancers.

**TABLE 1:** Sequence of the top 20 small molecules with highest docking scores.

Number	Name	Score
001	Specs_AJ-030/14523202	−6.8
002	Specs_AP-263/41670332	−6.429
003	Specs_AL-466/21162036	−6.344
004	Specs_AJ-030/14523202	−6.105
005	Specs_AK-778/41182454	−5.87
006	Specs_AT-057/43208217	−5.819
007	Specs_AE-641/10671041	−5.627
008	Specs_AF-399/13806211	−5.626
009	Specs_AK-968/41925468	−5.615
010	Specs_AK-918/12272295	−5.61
011	Specs_AN-979/41713880	−5.409
012	Specs_AE-848/37174093	−5.393
013	Specs_AN-698/40780603	−5.302
014	Specs_AE-562/12222184	−5.25
015	Specs_AK-968/13035148	−5.25
016	Specs_AN-919/14229193	−5.193
017	Specs_AN-465/40769611	−5.192
018	Specs_AO-022/43452701	−5.192
019	Specs_AK-968/41925169	−5.189
020	Specs_AN-698/41886126	−5.114

## Discussion

Recent evidence suggests that AQP4 expression may be linked to several diseases such as cancer (Papadopoulos and Saadoun, 2015; Zou et al., 2020; Lan et al., 2020). Nevertheless, precise mechanisms have yet to be fully elucidated. Current evidence has indicated that brain AQP4 is up-regulated in high grade tumors compared to low grade tumors or normal brain tissue, possibly contributing to cerebral edema (Saadoun et al., 2002; Warth et al., 2007). Other authors link AQP4 to the regulation of human glioma cells migration and invasion (Ding et al., 2010; Ding et al., 2011). Besides, various studies have confirmed the increased AQP4 expression in GBM, and promoting role of the down regulation of AQP4 in inducing glioblastoma cell apoptosis (Ding et al., 2013) has also been elucidated. All these suggest the involvement of AQP4 in malignant brain tumors and indicated that AQP4 could serve as a potential target for therapy of glioma. This study is the first to comprehensively examine AQP4 gene expression in human brain tumors available in the TCGA and GEO databases as well as its relationship to protein modulation, genetic alteration and other gene expression.

Mammals have been found to demonstrate 13 different aquaporins (AQP0-AQP12). AQP4 has been found to play a central growth in cancer development and progression (Lan et al., 2017). Some studies found that inhibition of AQP4 expression resulted in slowed tumor growth (Ding et al., 2011; Ding et al., 2013). Nicosia et al. (Nicosia et al., 2019) found that both CD4^+^ and CD8^+^ T cells expression AQP4. T cell proliferation, trafficking and activation were stunted as a result of AQP4 blockade. Furthermore, critical chemokine receptors known to mediate T cell migration were also affected by T cell inhibition. AQP4 targeting may be useful in eliminating aberrant immune responses in patients with various immune-related diseases. Our study further clarified the potential therapeutic effect of AQP4 in CNS cancers and its immune-related biological mechanisms.

Our previous research indicated that AQP4 could be was found in high concentrations in brain cancer tissues and that AQP4 could also impact the overall survival of cancer patients (Lan et al., 2020). However, the roles of AQP4 in immunity, as well as in various other brain cancers have not been identified systematically. Interestingly, ours investigation found that other tumors expressed AQP4 to a lower degree compared to brain cancers such as GBM and LGG, implicating AQP4 in brain cancer tumorigenesis. Different cancers were demonstrated to have distinct profiles of AQP4 expressions.

AQP4 was also found to have significant statistical correlations to microsatellite instability, tumor mutational burden, immune cell infiltration, protein phosphorylation and clinical prognosis across multiple tumors. Whether or not AQP4 exerts a tumor-suppressive or oncogenic effect warrants further investigation. Additionally, there is no widely accepted specific AQP4 inhibitor, and aquaporin inhibitors may be a novel class of anti-tumor agents. Currently no such inhibitors are available to date, and studies exploring small molecule inhibitors targeting AQP4 have been largely unsuccessful (Verkman et al., 2014; Papadopoulos and Saadoun, 2015). Screening for specific inhibitors of AQP4 may illuminate the design of novel mechanism-based therapies for brain tumors. Thus, the potential AQP4 inhibitors presented in this study that could serve as a basis for increased research interest and warrant more detailed exploration. Recognition of AQP4-specific inhibitors may open a new avenue for developing more specific targeted treatment for brain cancers. Our study provides robust evidence supporting AQP4 as a new candidate for brain cancer treatment.

## Conclusion

Despite the interesting implications associated with AQP4 and its significant potential as a potential therapeutic target, the mechanisms of its potential effects on tumorigenesis or tumor suppression remain elusive. The findings presented in this study clearly demonstrated that AQP4 should be further studied. However, it is noteworthy that the foundational basis of the views presented in this article is based solely on the bioinformatic technique. More studies should be directed toward clarifying the precise effects of AQP4 in various human cancers.

## Data Availability

The raw data supporting the conclusion of this article will be made available by the authors, without undue reservation.
